# Assessing 48 SNPs in Hypertensive Paediatric Patients and Young Adults with Review of Genetic Background of Essential Hypertension

**DOI:** 10.3390/children9081262

**Published:** 2022-08-21

**Authors:** Mirjam Močnik, Boris Zagradišnik, Nataša Marčun Varda

**Affiliations:** 1 Department of Paediatrics, University Medical Centre Maribor, Ljubljanska ulica 5, 2000 Maribor, Slovenia; 2 Laboratory of Medical Genetics, University Medical Centre Maribor, Ljubljanska ulica 5, 2000 Maribor, Slovenia; 3 Medical Faculty, University of Maribor, Taborska 8, 2000 Maribor, Slovenia

**Keywords:** obesity, hypertension, genetics, single-nucleotide polymorphism, child

## Abstract

Essential hypertension in paediatric patients and young adults is rising, mostly on account of obesity-related hypertension. Clinically, the difference between obese hypertensive and non-obese hypertensive individuals is evident; yet, the pathophysiology of essential and obesity-related hypertension is multifactorial, complex and not fully understood. The aim of our study was to obtain a comprehensive view of the clinical differences between obesity-related hypertension and hypertension in non-obese paediatric patients and young adults and to do genetic tests to possibly highlight some of the pathophysiological differences with a review of their genetic backgrounds. Four hundred and thirty-six hypertensive paediatric patients and young adults were included in the study, and a study of 48 single-nucleotide polymorphisms, using Kompetitive allele specific PCR, was conducted. The subjects were divided into 243 non-obese participants with hypertension and 193 obese participants with hypertension. The data for the clinical comparison of both groups were collected as well. The differences in some clinical and biochemical parameters were confirmed. Genetic tests showed a significant difference in one allele frequency between both groups in five SNPs: rs6232, rs6235, rs12145833, rs59744560 and rs9568856. In rs6235 and rs59744560, a direct effect of different allele states could be implied. Obesity-related hypertension at a young age differs from essential hypertension in those non-obese. The reported genetic differences could be important in understanding the complex pathophysiology of early-onset obesity-related hypertension and should be further evaluated.

## 1. Introduction

Childhood obesity is an increasing health problem worldwide, and its prevalence is still rising significantly in most countries [1]. In Slovenia, almost one-fifth of five-year-old children are overweight and almost 10% obese, similar to the other European countries [2]. Obesity plays a central role in hypertension, hyperinsulinemia, type 2 diabetes mellitus and hyperlipidaemia development, with an increased risk of atherosclerotic cardiovascular disease. Its aetiology is multifactorial [3].

It has been estimated that up to 30% of obese children suffer from hypertension. In addition, 56% of children and adolescents with persistently elevated blood pressure are found to be overweight [1]. The pathophysiology of essential hypertension is complex and not fully understood. The involvement of both, genetic and environmental factors and their interactions have been found to be important, and the same seems to be true for obesity-related hypertension. In the latter, different mechanisms have been proposed, including the sympathetic nervous system, some adipokines, hyperinsulinemia, inflammation and the renin-angiotensin-aldosterone system [4,5,6,7,8]. Although obesity is clearly associated with an increased prevalence of hypertension, many obese individuals do not develop hypertension [9]. In the last decade, investigations of the genetic components of both have brought some new knowledge. However, much remains to be done [8,10,11].

Continuing genetic studies, such as single-nucleotide polymorphisms (SNPs), copy number variations and genome-wide association studies (GWAS), have already been performed and seem promising in complex diseases such as hypertension. Simultaneously, studies of their functional effects and physiological consequences are needed to provide better biological insight [10,11].

In our study, we wanted to emphasize the differences between essential hypertension in non-obese and obesity-related hypertension in children and adolescents by comparing their clinical parameters. In addition, we focused on the research of 48 SNPs, searching the difference of occurrence between both groups. They were selected before genetic testing after a review of the current knowledge in the genetic background of essential hypertension in the young to provide well-chosen loci related to hypertension and obesity according to the possible pathophysiological mechanisms and potential etiologic pathways. Our study offers a unique perspective on obesity-related hypertension in the young with both clinical and genetic components.

## 2. Genetic Background of Essential Hypertension—Literature Review

Blood pressure is a quantitative trait that is highly variable and remains a major modifiable risk factor for cardiovascular diseases. Although the name “essential hypertension” implies that the cause is not known, this is not entirely true. Along with modifiable environmental factors, such as obesity, insulin resistance, aging, sedentary lifestyle, stress, etc., several genetic variations of genes that are overexpressed or under-expressed, as well as the intermediary phenotypes that they regulate, are known to be involved in blood pressure regulation [4].

The simplest form of DNA variation is the substitution of one single nucleotide for another—SNP. They occur at a frequency of approximately one in 1000 base pairs, and more than three million SNPs have been charted so far. The changes are believed to be stable and not deleterious to organisms, but they might produce diseases and may be responsible for the susceptibility of an individual to many common diseases, drug metabolism and genome evolution [12]. With the evolution of molecular testing, genome-wide association studies allow rapid scanning across the complete sets of DNA or genomes of many people to find the genetic variations associated with particular diseases. In Table 1, commonly known SNPs related to hypertension, obesity or metabolic syndrome are presented. Only variants, possibly involved in the pathological state, are listed. Monogenic hypertension genes of rare familial hypertensive or obesity syndromes were excluded. 

**Table 1:** Review of SNPs related to hypertension, obesity or metabolic syndrome. * Association observed in children.

Gene	SNPs	Related to	Reference
*ADCY9*	rs2531995	obesity	[12]
*AKTI*	rs1130214 rs10141867 rs33925946	metabolic syndrome	[13]
*APOA1/C3/A4/A5*	rs964184	metabolic syndrome	[14]
*APOA5*	rs2266788	metabolic syndrome	[14]
*APOC1*	rs4420638	metabolic syndrome	[14]
*ATP2B1*	rs2681492 rs2681472	hypertension	[15,16]
*BDNF*	rs10767664 * rs4923461 rs6265 * rs2030323 rs925946	obesity	[12,14,17]
*BRAP*	rs11065987	metabolic syndrome	[14]
*BUD13*	rs10790162	metabolic syndrome	[14]
*CACNB2*	rs11014166	hypertension	[15]
*CADM2*	rs13078807 rs17292179 *	obesity	[12,14,17,18]
*CASZ1*	rs880315	hypertension	[16]
*CDH13*	rs11646213	hypertension	[15]
*CDH17*	rs7006531	hypertension	[19]
*CETP*	rs173539	metabolic syndrome	[14]
*CSK*	rs1378942	hypertension	[15,16]
*CSK-ULK4*	rs6495122	hypertension	[15]
*CYP17A1*	rs11191548 rs1004467 rs12413409	hypertension	[15,16]
*eNOS*	rs1799983	hypertension, obesity	[20]
*ETV5*	rs9816226 * rs7647305 rs17295554 *	obesity	[12,14,17]
*EVX1/HOXA cluster*	rs11563582	hypertension	[19]
*FAIM2*	rs7138803	obesity	[12,14,17]
*FANCL*	rs887912	obesity	[12,14,17]
*FGF5*	rs16998073	hypertension	[16]
*FLJ35779-POC5*	rs2112347	obesity	[12]
*FRMD3*	rs115795127	hypertension	[19]
*FTO*	rs1558902 * rs1121980 * rs9939609 * rs8050136 * rs17817449 * rs12149832 * rs9930333 *	obesity	[12,14,17]
*GNAT2*	rs17024258	obesity	[12]
*GNPDA2*	rs10938397 *	obesity	[12,14,17]
*GPRC5B*	rs12444979 rs4780831 *	obesity	[12,14,17,18]
*GPR20*	rs78192203	hypertension	[19]
*HOXA*	rs6969780	hypertension	[19]
*HS6ST3*	rs7989336	obesity	[12]
*HNF4G*	rs4735692	obesity	[12]
*IGFBP3*	rs11977526	hypertension	[19]
*ITGA9*	rs155524	hypertension	[16]
*KCTD15*	rs29941 * rs11084753	obesity	[12,14,17]
*LLPH/TMBIM4*	rs113866309	hypertension	[19]
*LPL*	rs295	metabolic syndrome	[14]
*LRP1B*	rs2890652 rs1476250 *	obesity	[12,14,17,18]
*LRRN6C*	rs10968576	obesity	[12,14]
*MAP2K5*	rs2241423 rs4776970	obesity	[12,14,17]
*MC4R*	rs571312 * rs12970134 * rs17773430 * rs17782313	obesity	[12,14,17,18]
*MRPS22*	rs7638110	obesity	[18]
*MRPS33P4*	rs13041126	obesity	[12]
*MTCH2*	rs3817334 rs10838738 rs7120548 *	obesity	[12,14,17,18]
*MTHFR*	rs17367504	hypertension	[15]
*MTIF3*	rs4771122 rs1885989 *	obesity	[12,14,17,18]
*NEGR1*	rs2815752 * rs2568958 * rs2630397 *	obesity	[12,14,17,18]
*NRXN3*	rs10150332 * rs10483942 *	obesity	[12,14,17,18]
*NUDT3*	rs206936	obesity	[12,14,17]
*PAX5*	rs16933812	obesity	[18]
*PLCG1*	rs753381	metabolic syndrome	[14]
*PLCD3*	rs12946454	hypertension	[15]
*PLEKHA7*	rs381815	hypertension	[15]
*PLEKHG1*	rs62434120	hypertension	[19]
*PRDM8/FGF5*	rs16998073	hypertension	[15]
*PRKD1*	rs11847697	obesity	[12,14,17]
*PTBP2*	rs1555543	obesity	[12,14,17]
*QPCTL*	rs2287019	obesity	[12,14,17]
*RBJ*	rs713586	obesity	[12,17]
*RPL27A*	rs4929949 rs7925000 *	obesity	[12,14,17,18]
*RPTOR*	rs7503807	obesity	[12]
*SEC16B*	rs543874 * rs574367 rs516636 * rs10159282 * rs10913469	obesity	[12,14,17,18]
*SH2B1*	rs7359397 * rs7498665	obesity	[12,14,17]
*SH2B3*	rs653178 rs3184504	hypertension	[15]
*SLC39A8*	rs13107325 rs2165265 *	obesity	[12,14,17,18]
*STK39*	rs6749447 rs3754777	hypertension	[15,21]
*TARID/TCF21*	rs76987554	hypertension	[19]
*TBX3-TBX5*	rs2384550	hypertension	[15]
*TFAP2B*	rs987237 *	obesity	[12,14,17]
*TMEM160*	rs3810291	obesity	[12,14]
*TMEM18*	rs2867125 * rs6548238 * rs7561317 *	obesity	[12,14,17]
*TNNI3K*	rs1514175	obesity	[12,14,17]
*ULK4*	rs9815354 rs7651190 rs7372217	hypertension	[15,19]
*ZNF259*	rs2075290	metabolic syndrome	[14]
*ZNF608*	rs4836133 rs4421098 *	obesity	[12,14,17,18]
*ZNF652*	rs16948048	hypertension	[15]
*ZZZ3*	rs17381664	obesity	[19]

## 3. Materials and Methods

Our study included children, adolescents and young adults with hypertension that were treated at our department, either in an outpatient clinic or were hospitalized for further diagnostic tests. Blood sampling for genetic tests was conducted based on the presence of hypertension—blood pressure ≥95th percentile measured on three different occasions [22]. Parents or legal guardians were obliged to sign the informed consent form. We collected samples from 436 participants (289 male, 147 female) that were later divided into obese and non-obese according to International Obesity Task Force (IOTF) reference [23], resulting in 243 non-obese and 193 obese participants with hypertension aged 2–21 years. Thirty-two children were under the age of 6 and actually considered infants. They were included in the study because of the emphasized effect of genetic background at an early age.

Genetic tests were conducted by KBioscience or LGC Genomics SNP genotyping service using Kompetitive Allele Specific PCR or KASP. It is one of the SNP genotyping platforms, and several aspects need to be considered when selecting the most suitable genotyping platform for a specific application. It is based on allele-specific oligo extension and fluorescence energy transfer for signal generation. One of the most attractive features of KASP is its cost-effectiveness and the duration of the synthesis of the assay. It is also flexible in applications that require small to moderate numbers of markers, such as a quality control analysis, quantitative trait loci fine mapping and others [24]. We selected 48 SNPs that were associated with hypertension and obesity according to various data, with the reported occurrence in the young of European ancestry. SNPs were also selected by their assumed functional role to include different hypertension development processes. Therefore, the following SNPs were included: rs2004776, rs13107325, rs1800629, rs2276047, rs3753519, rs17782313, rs2287019, rs3842759, rs12444979, rs1514175, rs13078807, rs1424233, rs1805081, rs10508503, rs7138803, rs2116830, rs4994, rs7498665, rs6548238, rs16933812, rs753381, rs7638110, rs9568856, rs1993709, rs10938397, rs6232, rs6235, rs7132908, rs3758539, rs1862513, rs11191548, rs10913469, rs9299, rs12145833, rs17150703, rs61866610, rs173539, rs964184, rs10767664, rs59744560, rs1570070, rs4305, rs9930506, rs2241423, rs10150332, rs11847697, rs1555543 and rs116454156. KASP genotyping was carried out in a 96-well plate format to include both alleles in the chosen SNPs.

In addition, anthropometric and laboratory data have been collected during their hospital stay to compare our subjects in a clinical view. Anthropometric measurements were done at admittance (height, weight, body mass index (BMI) and waist and hip circumference) and were performed by trained and experienced personnel. The 24-h ambulatory blood pressure monitoring using age-appropriate cuffs was done to confirm the diagnosis of hypertension. The laboratory analysis included a complete blood count, blood levels of glucose, urea, creatinine, cystatin C, uric acid, sodium, potassium, chlorides, calcium, magnesium, phosphate, cholesterol, triglycerides, high-density lipoprotein (HDL), low-density lipoprotein (LDL), homocysteine, lipoprotein(a), apolipoprotein A-1 (APOA1), apolipoprotein B (APOB) and urine testing with the urine albumin/creatinine ratio. Urine testing was done with one urine sample, where specific weight was determined with semiquantitative measurements of the glucose, bacteria, leukocytes, nitrates, pH, methyl ketones and bilirubin. For all gathered data, including the genotyping results, statistics was done with SPSS Statistics 20. To determine whether there was a significant difference between the frequencies of the SNPs in both groups, the chi-square test was used. For clinical comparison, we used the Kruskal–Wallis and Mann–Whitney *U* test. *p* < 0.05 was considered statistically significant. The study was approved by the national ethic committee (UKC-MB-98/11/11).

## 4. Results

Group 1 consisted of 243 non-obese hypertensive and Group 2 of 193 obese hypertensive children, adolescents and young adults. In Table 2, the clinical characteristics of both groups with their comparison are presented. The data are given as the mean value (standard deviation) (minimum–maximum) and *p*-value. All the laboratory data in Table 1 resulted from a blood serum analysis. The urine analysis data showed no significant results and are not shown.

The genotyping results are presented in Table 3. All chosen SNPs with associated genes are shown with Pearson’s chi-square and the *p*-value. For each SNP, both scenarios with a dominant allele were assumed. Statistically important differences were found in five SNPs: rs6232 and rs6235 in the gene for proprotein convertase subtilisin/kexin type 1 (PCSK1), rs12145833 in the gene for serologically defined colon cancer antigen 8 (SDCCAG8), rs59744560 in the gene for nicotinamide phosphoribosyltransferase (NAMPT) and rs9568856 in the gene for olfactomedin 4 (OLFM4).

Finally, we divided the subjects five times according to their alleles and compared the clinical data between groups with different alleles in SNPs that were found statistically important. The goal was to detect if different SNPs affect the clinical presentation. The Mann–Whitney *U* test was used for comparisons, and only *p*-values of the results are presented in Table 4. In rs6235, the systolic pressure and serum glucose differed between allele carriers and in rs59744560 serum leukocyte and platelets counts.

## 5. Discussion

Two clinically different groups of hypertension patients were studied by anthropometric measurements and laboratory findings. Significantly higher weight, BMI and waist and hip circumference were expected in Group 2 but not significantly lower height, since obese growing children and adolescents tend to grow taller [25]. The reason for this anomaly is probably due to the lower mean age in obese children, which could indicate that obese children develop hypertension earlier than non-obese hypertensive patients. However, obesity-related hypertension depends on the BMI, and the risk for hypertension in children and adolescents has proven to increase with the increase of the BMI values [26,27].

Physiologically, blood pressure gradually increases in children, which is the reason the mean values in Group 1 are higher, because the average age is also higher in the group. Therefore, both systolic and diastolic pressure are lower in Group 2. However, the effect of obesity is challenging to estimate, because all patients had proven hypertension.

It has been estimated that 10–25% of obese children may have impaired glucose tolerance, and 4% may have silent type 2 diabetes mellitus [26]. In our study, higher values of glucose were observed in Group 2, though not reaching statistical significance.

Next, higher values of both urea and creatinine were observed in Group 1, though reaching significance only for creatinine. For the latter, it has been shown to be a predictive factor of poor outcomes in obesity-related glomerulopathy, but only a minority of those obese develop such a severe injury [28]. Cystatin C, an early renal injury marker, is similar in both groups, which supports the assumption that the renal function does not differ to such an extent in our subjects.

The association between uric acid and cardiovascular diseases in adults has been shown in many studies. It has also been confirmed in obese children and adolescents [29]. Recent studies suggest that uric acid leads to endothelial dysfunction by stimulating vascular smooth muscle proliferation and a decrease in nitric oxide production [29]. This could be partly a reason for the hypertension development in Group 2, which has a much higher mean uric acid level. In addition, cross-sectional analyses of general paediatric populations showed a possible association between diastolic blood pressure and both uric acid concentration and xanthine oxidase activity [30].

Observed electrolyte disturbances included higher sodium and lower potassium, chlorides and phosphate levels in Group 1. However, electrolyte homeostasis depends on multiple factors, and we cannot even imply which are responsible for the differences. Otherwise, the relationship between the sodium intake and blood pressure is well-established [7]. Lower phosphate levels were also already associated with the risk of hypertension [31] and could be characteristic for hypertension in those non-obese. However, obesity also causes mineralocorticoid receptor activation independent of aldosterone or angiotensin II. The activation of the renin-angiotensin-aldosterone system is, in part, a consequence of the sympathetic nervous system function. In obesity, its mechanisms are not fully elucidated but may involve leptin and activation of the brain melanocortin system [32]. Gorzelniak et al. showed a difference between renin-angiotensin system genes regulation in human obesity and hypertension in comparison to lean or obese normotensive individuals [33].

With the epidemic of obesity, dyslipidaemia has emerged as an additional characteristic of obese individuals. Our study confirmed a significant rise in LDL and triglycerides and a decrease in HDL in obese participants. Hyperlipidaemia can affect vascular endothelial function and impair some regulatory properties, thus initiating the atherosclerotic process [34] and could promote obesity-related hypertension development. Our results also showed slightly higher homocysteine levels in non-obese participants. In some studies of overweight and obese children, its importance was not confirmed [35]. High lipoprotein(a) is a predictor of early atherosclerosis that is independent of other risk factors, such as hypertension and obesity [36], as confirmed by our results. Apolipoprotein A1 and apolipoprotein B differences are affected by obesity. However, other studies report similar values when comparing their levels with those non-obese [37].

The complete blood count showed various significant differences between both groups. In part, that could be explained by changes in the peripheral blood in obese, otherwise healthy, individuals, as reported by Trellakis et al. Their study supported enhanced apoptosis and neutrophil activity in the peripheral blood of healthy overweight subjects [38]. Vuong et al. noticed similar differences and attributed them to a low-grade inflammatory state in those obese due to insulin resistance and chronic activation of the immune system [39].

In the second part of our study, some SNPs, the genomic markers that are genotyped and with which disease associations are tested [10], were investigated. We already implied that numerous genetic tests are still needed to get closer to understanding the genetics of obesity and hypertension. More than 300 SNPs have been identified in association with adiposity traits [40]. There are many factors that influence the blood pressure response to weight change, suggesting that many genetic variants may affect the sensitivity of the mentioned response. The latter is based on the observation that patients carrying certain SNPs related to hypertension and obesity seem more likely to benefit from a diet for blood pressure control than others [41]. In our study, we focused on 48 SNPs that might be significant in the connection of obesity-related hypertension because of the potential pathophysiological mechanism. We were able to prove some statistically significant SNP occurrence differences between both groups that are discussed below.

*PCSK1* gene encodes the prohormone convertase 1/3 enzyme (PC 1/3) that converts prohormones into functional hormones, which regulate the energy metabolism. The enzyme is expressed in neuroendocrine cells. Some studies of both loci presented in Table 2 have been conducted and gave ambiguous results. Benzinou et al. found both rs6232 and rs6235 to be consistently associated with common obesity. Their functional analysis showed a significant impairment of the PC 1/3 protein catalytic activity [42]. On the contrary, Kilpeläinen et al. did not find rs6232 and rs6235 to contribute to common obesity. The exception was the association of rs6232 and obesity in the younger [43]. However, Löffler et al. confirmed the role of rs6232 in *PCSK1* by sequencing the gene as a polygenic risk variant for childhood obesity [44]. Our results also support the association between SNPs in *PCSK1* with obesity and obesity-related hypertension in the young; thus, the potential neuroendocrine involvement in obesity-related hypertension development in these patients.

Genetic variants in the *SDCCAG8* gene have been associated with early-onset obesity in Scherag et al. Loci have been derived from the GWAS analysis, and additional tests showed consistent association of the *SDCCAG8* variation with obesity. The data on this gene is generally limited, and its involvement in body weight regulation is not clear. A high transcript abundance of the protein was found in the hypothalamus, pituitary and adrenals that indicates the hormonal axis as the reason for its body regulation involvement. Its role in centrosomal organization has been suggested. Additionally, it is considered to be a naturally occurring autoantigen and is ubiquitously expressed in the thymus, small intestine, colon mucosa, liver and brain, among other tissues [45]. According to variance differences between both groups, we assume that the possible endocrine role of *SDCCAG8* could contribute to the development of obesity-related hypertension.

Visfatin or the product of *NAMPT* gene is mainly produced and secreted by visceral fat. It is a multifaceted molecule with proinflammatory cytokine and enzymatic properties. It enhances macrophage-induced inflammation, and this effect may result in a variety of alterations that may be involved in atherogenesis and other pathogenic mechanisms that occur early in childhood obesity. There is strong evidence of visfatin increase in obesity in adults and children; however, the genetic polymorphism evidence is scarce. Belo et al. found no significant differences in the study of two SNPs in the *NAMPT* gene in allele frequency distributions when compared to healthy controls [46]. Our results found the association of a gene polymorphism and its role in obesity-related hypertension, though we studied another SNP.

*OLFM4* encodes a glycoprotein that facilitates cell adhesion on the cell surface. There have been many studies of gene polymorphisms in the connection to various cancers. Polymorphism rs9568856 has been associated with obesity, especially during childhood. Some observations link it to gut microflora and to a relationship between the microbiome and obesity risk; however, its function is not well-understood [47]. Our results confirmed its potential role in obesity and, with unknown mechanism, a potential role in hypertension development in those obese.

In all other investigated genetic factors, no differences between our two groups of hypertensive patients have been found, implicating that they are most probably not important in their distinction.

Obesity and hypertension are both multifactorial diseases, and their aetiology cannot be explained by solely one factor. However, the effect of a single gene defect in an individual is yet to be determined. Therefore, we divided our subjects according to their allele status in SNPs, found to be statistically different. Interestingly, we found important differences in two SNPs, showing that different alleles could have an impact on systolic pressure and serum glucose in rs6235 and on the leukocyte and platelet counts in rs59744560. Otherwise, there were no other observed differences, which is consistent with the genetic basis of both diseases.

The limitations of our study are a relatively low number of participants, and therefore, we could not subdivide them according to age group or sex. Additionally, due to the very wide range of genetic defects possible in hypertension development, 48 SNPs are insufficient to cover a significant proportion of the genetics of essential hypertension.

Obesity-related hypertension is rising in children, adolescents and young adults. Both obesity and hypertension alone are associated with increased CVD risk, often continuing from childhood into adulthood, increasing the prevalence of CVD and related morbidity and mortality. The proposed genetic factors should be reviewed to provide a better understanding of the development of obesity-related hypertension and a targeted diagnostic and future treatment approach [48].

## 6. Conclusions

Our study offers a new perspective on obesity-related hypertension in comparison to essential hypertension in non-obese young patients with comprehensive clinical comparison and genetics, though with a relatively small number of subjects. We found some clinical and genetic differences, which might be clinically important—specifically, *PCSK1*, *SDCCAG8*, *NAMPT* and *OLFM4*. We were able to imply a possible direct phenotypic effect of different alleles in some SNPs. Our results should be confirmed in other prospective studies and the aetiology of obesity-related hypertension further explored. A review of the genetics of essential hypertension in children was added and could offer a starting point for further research.

## Figures and Tables

**Table 2:** Clinical characteristics and comparison of the subjects. The data are given as the mean value (standard deviation) (minimum–maximum) and *p*-value. BMI—body mass index, HDL—high-density lipoprotein, LDL—low-density lipoprotein, Lp(a)—lipoprotein (a), APOA—apolipoprotein A1, APOB—apolipoprotein B, MCV—mean corpuscular volume, MCH—mean corpuscular haemoglobin and MCHC—mean corpuscular haemoglobin concentration.

Clinical Parameter	Group 1	Group 2	*p*-Value
Age	14.71 (3.7) (2–21)	12.83 (4.2) (2–19)	<0.001
Height (m)	1.67 (0.2) (0.98–2.04)	1.61 (0.2) (0.95–1.93)	<0.001
Weight (kg)	64.82 (17.6) (14.5–99)	80.8 (27.4) (18–157)	<0.001
BMI (kg/m^2^)	22.43 (3.1) (13.5–27.8)	29.93 (5.3) (19.1–48)	<0.001
Waist circumference (cm)	79.81 (11) (46–112)	95.29 (15.8) (59–150)	<0.001
Hip circumference (cm)	92.9 (12) (54–127)	104.9 (16.7) (63–159)	<0.001
Systolic pressure (mmHg)	130.5 (10.1) (98–156)	128.5 (11) (101–166)	0.021
Diastolic pressure (mmHg)	73.93 (8.5) (52–107)	72.25 (8.8) (53–100)	0.027
Glucose (mmol/L)	4.7 (0.6) (3.5–7.1)	4.8 (0.5) (3.6–7)	0.037
Urea (mmol/L)	4.38 (1) (2.2–7.6)	4.28 (0.9) (2.3–8.1)	0.385
Creatinine (µmol/L)	69.3 (20.6) (25–237)	60.64 (17) (22–120)	<0.001
Cystatin C (mg/L)	0.78 (0.1) (0.47–1.04)	0.8 (0.1) (0.57–1.15)	0.489
Uric acid (µmol/L)	285.3 (66.3) (139–509)	313 (78) (105–551)	<0.001
Sodium (mmol/L)	138.3 (1.8) (133–143)	137.8 (1.9) (133–142)	0.001
Potassium (mmol/L)	4.14 (0.3) (3.37–5.48)	4.27 (0.3) (3.66–5.36)	<0.001
Chlorides (mmol/L)	102.3 (2.3) (93–109)	102.9 (2.2) (96–109)	0.009
Calcium (mmol/L)	2.39 (0.2) (1.15–4.35)	2.41 (0.2) (2.15–4.94)	0.528
Magnesium (mmol/L)	0.81 (0.1) (0.65–1.22)	0.81 (0.1) (0.65–1.06)	0.720
Phosphate (mmol/L)	1.24 (0.2) (0.63–1.91)	1.32 (0.3) (0.79–1.94)	0.003
Cholesterol (mmol/L)	4.34 (0.9) (2.5–7.55)	4.45 (0.9) (1.76–6.85)	0.077
Triglycerides (mmol/L)	1.02 (0.6) (0.19–3.36)	1.24 (0.7) (0.21–3.9)	<0.001
HDL (mmol/L)	1.4 (0.3) (0.61–2.82)	1.23 (1.5) (0.54–2.28)	<0.001
LDL (mmol/L)	2.53 (0.7) (1–4.6)	2.79 (0.8) (0.7–5.2)	0.001
Homocysteine (µmol/L)	10.92 (7) (3.5–77.5)	9.74 (4.1) (3.8–36.1)	0.016
Lp(a) (µmol/L)	0.21 (0.3) (0.02–1.46)	0.2 (0.3) (0.02–1.61)	0.622
APOA1 (µmol/L)	1.5 (0.3) (0.95–2.51)	1.38 (0.3) (0.82–2.5)	<0.001
APOB (µmol/L)	0.75 (0.2) (0.32–1.51)	0.81 (0.2) (0.23–1.32)	0.004
Leukocyte (×10^9^)	6.55 (1.8) (3.1–14.18)	7.18 (2.1) (3.5–24.17)	<0.001
Erythrocyte (×10^9^)	5.06 (0.4) (4.1–6.7)	5.16 (0.4) (4.2–6.1)	0.005
Haemoglobin (g/L)	146.7 (12.8) (112–177)	144 (13.2) (116–176)	0.010
Haematocrit	0.42 (0.03) (0.35–0.5)	0.42 (0.03) (0.34–0.5)	0.116
MCV (fl)	83.92 (6.4) (73–93.7)	81.17 (4.9) (68–93.4)	<0.001
MCH (pg)	29 (1.5) (24.8–32.2)	27.8 (1.9) (21–31.3)	<0.001
MCHC (g/L)	346 (10.2) (319–370)	342.9 (10) (302–367)	0.003
Platelets (×10^9^)	263.3 (61.3) (96–497)	290 (59.9) (162–457)	<0.001

**Table 3:** Chi-square test for each SNP. The first Pearson chi-square value for each SNP represents the scenario when the first allele is dominant and the second when the second allele is dominant. PCS—Pearson’s chi-square, *AGT*—angiotensinogen, *SLC39A8*—solute carrier family 39 member 8, *TNF ALPHA*—tumour necrosis factor alpha, *INPPL1*—inositol polyphosphate phosphatase like 1, *HSD11B1*—hydroxysteroid 11-beta dehydrogenase 1, *MC4R*—melanocortin 4 receptor, *QPCTL*—glutaminyl-peptide cyclotransferase like, *INS-IGF2*—insulin-insulin like growth factor 2, *GPRC5B*—G protein-coupled receptor class C group 5 member B, *TNNI3K*—troponin I3, cardiac type, interacting kinase, *CADM2*—cell adhesion molecule 2, *MAF*—musculoaponeurotic fibrosarcoma oncogene homolog bZIP transcription factor, *NPC1*—Niemann-Pick C1 intracellular cholesterol transporter 1, *PTER*—phosphotriesterase related, *FAIM2*—Fas apoptotic inhibitory molecule 2, *KCNMA1*—potassium calcium-activated channel subfamily M alpha 1, *ADRB3*—adrenoceptor beta 3, *SH2B1—SH2*-domain containing mediators family adaptor protein 1, *TMEM18*—transmembrane protein 18, *PAX5*—paired box 5, *PLCG1*—phospholipase C gamma 1, *MRPS22*—mitochondrial ribosomal protein S22, *OLFM4*—olfactomedin 4, *NEGR1*—neuronal growth regulator 1, *GNPDA2*—glucosamine-6-phosphate deaminase 2, *PCSK1*—proprotein convertase subtilisin/kexin type 1, *AIM2*—absent in melanoma 2, *RBP4*—retinol binding protein 4, *RETN*—resistin, *CYP17A1*—cytochrome P450 family 17 subfamily A member 1, *SEC16B*—SEC16 homolog B, endoplasmic reticulum export factor, *HOXB5*—homeobox B5, *SDCCAG8*—serologically defined colon cancer antigen 8, *TNKS/MSRA*—tankyrase/methionine sulfoxide reductase A, *GPR120*—free fatty acid receptor 4, *ZPR1*—zinc finger protein ZRP1, *BDNF*—brain-derived neurotrophic factor, *NAMPT*—nicotinamide phosphoribosyltransferase, *IGF2R*—insulin-like growth factor 2 receptor, *ACE*—angiotensin I converting enzyme, *FTO*—alpha-ketoglutarate dependent dioxygenase, *MAP2K5*—mitogen-activated protein kinase kinase 5, *NRXN3*—neurexin 3, *PRKD1*—protein kinase D1, *PTB2*—polypyrimidine tract binding protein 1 and *CEPT*—cholesteryl ester transfer protein.

Gene	SNP	PCS Value	*p*-Value	Gene	SNP	PCS Value	*p*-Value
*AGT*	rs2004776	2.601	0.272	*near GNPDA2*	rs10938397	3.859	0.145
		0.142	0.932			4.081	0.130
*SLC39A8*	rs13107325	4.529	0.104	***PCSK1-S690T***	**rs6232**	**7.121**	**0.028**
		3.938	0.140			2.588	0.274
*TNF ALPHA*	rs1800629	3.026	0.220	***PCSK1-N221D***	**rs6235**	**9.156**	**0.010**
		3.477	0.176			1.356	0.508
*INPPL1*	rs2276047	3.050	0.218	*AIM2*	rs7132908	3.178	0.204
		2.178	0.336			2.339	0.310
*HSD11B1*	rs3753519	3.865	0.145	*RBP4*	rs3758539	0.443	0.801
		1.341	0.512			0.091	0.955
*MC4R*	rs17782313	0.600	0.741	*RETN*	rs1862513	0.492	0.782
		0.524	0.770			0.152	0.927
*QPCTL*	rs2287019	1.642	0.440	*near CYP17A1*	rs11191548	2.306	0.316
		0.480	0.787			3.593	0.166
*INS-IGF2*	rs3842759	0.894	0.640	*SEC16B*	rs10913469	0.575	0.750
		0.828	0.661			2.360	0.307
*GPRC5B*	rs12444979	2.221	0.329	*HOXB5*	rs9299	0.601	0.741
		4.811	0.090			0.872	0.647
*TNNI3K*	rs1514175	0.215	0.898	***SDCCAG8***	**rs12145833**	4.875	0.087
		0.290	0.865			**9.515**	**0.009**
*CADM2*	rs13078807	2.109	0.348	*TNKS/MSRA*	rs17150703	7.732	0.421
		3.580	0.167			3.720	0.156
*near MAF*	rs1424233	1.759	0.415	*GPR120-R67C*	rs61866610	1.360	0.507
		2.341	0.310			1.098	0.295
*NPC1*	rs1805081	0.232	0.890	*GPR120-R270H*	rs116454156	1.098	0.295
		0.737	0.692			0.193	0.979
*near PTER*	rs10508503	1.826	0.401	*ZPR1*	rs964184	1.086	0.581
		2.359	0.308			0.167	0.920
*FAIM2*	rs7138803	2.207	0.332	*BDNF*	rs10767664	1.619	0.445
		2.581	0.275			2.164	0.339
*KCNMA1*	rs2116830	1.155	0.561	***NAMPT***	**rs59744560**	3.683	0.159
		1.572	0.456			**6.315**	**0.043**
*ADRB3*	rs4994	3.148	0.207	*IGF2R*	rs1570070	0.022	0.989
		5.469	0.065			0.001	1.000
*SH2B1*	rs7498665	1.162	0.559	*ACE*	rs4305	1.165	0.559
		0.180	0.914			0.791	0.852
*TMEM18*	rs6548238	2.385	0.304	*FTO*	rs9930506	5.068	0.079
		0.479	0.787			1.186	0.756
*PAX5*	rs16933812	2.336	0.311	*MAP2K5*	rs2241423	1.652	0.438
		0.848	0.655			1.220	0.748
*PLCG1*	rs753381	1.129	0.569	*NRXN3*	rs10150332	3.012 1.147	0.222 0.766
		4.479	0.214				
*MRPS22*	rs7638110	0.888	0.642	*PRKD1*	rs11847697	0.601	0.741
		1.356	0.716			0.117	0.943
***OLFM4***	**rs9568856**	**8.992**	**0.011**	*PTB2*	rs1555543	0.563	0.755
		2.270	0.518			0.958	0.811
*near NEGR1*	rs1993709	0.462	0.794	*near CEPT*	s173539	0.989	0.610
		0.283	0.963			1.023	0.599

**Table 4:** *p*-values for clinical data comparison between groups with different alleles within SNPs: BMI—body mass index, HDL—high-density lipoprotein, LDL—low-density lipoprotein, Lp(a)—lipoprotein (a), APOA1—apolipoprotein A1, APOB—apolipoprotein B, MCV—mean corpuscular volume, MCH—mean corpuscular haemoglobin and MCHC—mean corpuscular haemoglobin concentration.

Clinical Parameter	rs6232	rs6235	rs12145833	rs59744560	rs9568856
Age	0.902	0.169	0.960	0.545	0.078
Height (m)	0.736	0.090	0.412	0.939	0.815
Weight (kg)	0.268	0.826	0.135	0.302	0.110
BMI (kg/m^2^)	0.123	0.324	0.079	0.157	0.148
Waist circumference (cm)	0.271	0.633	0.257	0.414	0.450
Hip circumference (cm)	0.309	0.546	0.322	0.698	0.285
Systolic pressure (mmHg)	0.076	**0.022**	0.409	0.777	0.892
Diastolic pressure (mmHg)	0.492	0.122	0.638	0.685	0.454
Glucose (mmol/L)	0.641	**0.020**	0.736	0.135	0.309
Urea (mmol/L)	0.155	0.185	0.868	0.572	0.814
Creatinine (µmol/L)	0.629	0.199	0.620	0.824	0.436
Cystatin C (mg/L)	0.387	0.923	0.693	0.685	0.839
Uric acid (µmol/L)	0.118	0.520	0.537	0.458	0.761
Sodium (mmol/L)	0.199	0.155	0.888	0.442	0.501
Potassium (mmol/L)	0.856	0.730	0.833	0.786	0.473
Chlorides (mmol/L)	0.236	0.319	0.262	0.808	0.330
Calcium (mmol/L)	0.319	0.664	0.945	0.122	0.978
Magnesium (mmol/L)	0.133	0.294	0.556	0.723	0.199
Phosphate (mmol/L)	0.816	0.510	0.884	0.199	0.900
Cholesterol (mmol/L)	0.204	0.235	0.401	0.488	0.652
Triglycerides (mmol/L)	0.885	0.394	0.737	0.579	0.055
HDL (mmol/L)	0.213	0.316	0.957	0.595	0.108
LDL (mmol/L)	0.407	0.599	0.625	0.319	0.909
Homocysteine (µmol/L)	0.945	0.271	0.290	0.772	0.503
Lp(a) (µmol/L)	0.762	0.891	0.523	0.344	0.637
APOA1 (µmol/L)	0.056	0.899	0.984	0.317	0.369
APOB (µmol/L)	0.672	0.400	0.675	0.943	0.889
Leukocyte (×10^9^)	0.446	0.857	0.715	**0.037**	0.854
Erythrocyte (×10^9^)	0.097	0.329	0.480	0.062	0.498
Haemoglobin (g/L)	0.694	0.056	0.772	0.691	0.964
Haematocrit	0.683	0.054	0.729	0.606	0.835
MCV (fl)	0.062	0.062	0.159	0.307	0.473
MCH (pg)	0.154	0.166	0.170	0.217	0.330
MCHC (g/L)	0.913	0.374	0.513	0.519	0.781
Platelets (×10^9^)	0.889	0.105	0.636	**0.004**	0762

## Data Availability

All the data are available from the corresponding author upon reasonable request.
